# Advancements in Retinoblastoma Treatment: Unraveling the Potential of Intravitreal Chemotherapy

**DOI:** 10.7759/cureus.53012

**Published:** 2024-01-26

**Authors:** Shafiq Tanveer, Fahad Zafar, Hafsa Bibi, Hamza Haroon, Obaid Ahmad, Muhammad Shahid Iqbal, Zarafshan Zakir, Maryum Khilji, Safina Tanveer, Rao E Hassan

**Affiliations:** 1 Ophthalmology, Khyber Teaching Hospital MTI, Peshawar, PAK; 2 Surgery, Ayub Teaching Hospital, Abbottabad, PAK; 3 Ophthalmology, Hayatabad Medical Complex MTI, Peshawar, PAK; 4 Emergency Medicine, District Headquarter Hospital MTI, Bannu, PAK; 5 Internal Medicine, Khyber Teaching Hospital MTI, Peshawar, PAK; 6 Surgery, Khyber Teaching Hospital MTI, Peshawar, PAK; 7 Orthopaedics and Trauma, Khyber Teaching Hospital MTI, Peshawar, PAK

**Keywords:** retinoblastoma, chemoreduction, intra-vitreal chemotherapy, targeted therapies, ocular toxicity, intravitreal melphalan, treatment outcomes, global collaboration, advanced therapies, pediatric cancer

## Abstract

Retinoblastoma necessitates urgent attention due to its potential fatality if untreated. Multiple treatment options are available and should be employed according to size, location, and the extent of dissemination. This review emphasizes the need for increased awareness, advanced diagnostic tools, and innovative treatment approaches, especially intravitreal chemotherapy (IVitC) to address the diverse manifestations and aggressive nature of retinoblastoma. Timely diagnosis and commitment to treatment are pivotal, as delays and reluctance to undergo enucleation contribute to unfavorable outcomes. The evolving treatment landscape, spanning from traditional interventions to modern targeted therapies such as intravitreal melphalan, holds promise for improved outcomes. While the intravitreal approach presents challenges, ongoing research aims to establish its definitive role in retinoblastoma treatment. In the treatment of retinoblastoma, IVitC raises considerations about side effects. The risk of tumor spread beyond the eye is rare, emphasising the potential of IVitC in carefully selected cases. Intravitreal injections exhibit fewer local adverse effects compared to intra-arterial chemotherapy, with careful measures reducing significant ocular complications. The evaluation of ocular toxicity, particularly with melphalan, underscores the importance of a nuanced approach to achieve the right balance between therapeutic efficacy and ocular safety. This comprehensive analysis of studies on IVitC and its ocular and systemic complications provides valuable insights for enhanced patient care. The review concludes with a focus on balancing safety and efficacy in local chemotherapeutic drugs, highlighting the need for thoughtful measures and continued research to optimise treatment modalities globally.

## Introduction and background

Retinoblastoma, the prevailing malignant eye tumor in children, requires significant consideration due to its aggressive nature and the potential for fatality if left unattended. The reported incidence ranges from one in 15,000 to one in 18,000 live births, emphasizing the need for effective management [1]. Despite being less frequent than uveal melanoma, retinoblastoma poses distinctive challenges by mimicking over 25 other conditions in young patients, adding complexity to precise diagnosis. Fortunately, the disease does not exhibit any discernible racial or gender preference [2].

Worldwide, approximately 25-35% of cases impact both eyes, with diagnoses for bilateral cases typically established before the age of 12 months and for unilateral cases around the age of 24 months [3]. To guide treatment decisions, the International Classification of Retinoblastoma categorizes cases based on tumor size, location, and the extent of dissemination. This classification system encompasses five distinct groups ranging from small, isolated tumors (Group A) to cases that require immediate enucleation (Group E). The categorization includes retinoblastoma up to 3 mm (Group A), retinoblastoma larger than 3 mm, located in the macula, or showing minor subretinal fluid (Group B), retinoblastoma with localized seeds (Group C), retinoblastoma with diffuse seeds (Group D), and massive retinoblastoma necessitating enucleation (Group E) [4,5].

The aggressive characteristics and diverse manifestations of retinoblastoma pose challenges in achieving precise diagnoses, highlighting the crucial need for increased awareness, enhanced diagnostic tools, and advanced treatment approaches.

Epidemiology

Retinoblastoma is a rare pediatric cancer in the United States, observed in only 6% of children under the age of five, who are diagnosed with cancer. The frequency, averaging about 12 cases per million children aged 0-4, has remained consistent over the past three decades, aligning with statistics observed in Europe. Notably, it shows no gender or laterality preference, impacting both eyes and genders equally over time [6]. The global prevalence of retinoblastoma, as illustrated by prognostic models, is concerning. Asia bears the highest burden at 53%, followed by Africa (29%), Latin America (8%), North America (3%), and Europe (6%) [7]. This uneven distribution results in a worldwide survival rate of under 30%, sharply contrasting with the nearly 100% observed in high-income countries (HICs) where the illness is considered treatable [8].

Highlighting the challenges faced by developing nations, the data show a worrying reality. In countries with financial constraints, the average survival rate is around 40% (ranging from 23% to 70%). On the other hand, upper-middle-income countries have slightly better rates at 79% (ranging from 54% to 93%) [7]. Notably, Malaysia faces a troubling situation where 55% of cases involving retinoblastoma in children see the cancer spreading beyond the eye. This is mainly due to late diagnoses and a significant number of people stopping their treatment [9]. These concerning statistics underscore the importance of diagnosing the disease early and sticking to the treatment plan to improve survival rates.

Unfavorable outcomes are influenced by both delays in the initial diagnosis and reluctance to undergo enucleation, despite its potential life-saving benefits [10,11]. Unfortunately, the stigma surrounding enucleation dissuades many families from pursuing this essential treatment, ultimately facilitating the progression of the tumor beyond the eye.

Treatment options in retinoblastoma: an overview

Over the last three decades, there has been a significant transformation in the approach to managing retinoblastoma. The focus has shifted beyond mere survival to preserving patients' eyes, vision, and overall well-being, with the aim of minimizing the treatment burden [12]. Early detection of tumors and advancements in management techniques have contributed to a remarkable improvement in survival rates [13]. A comprehensive study covering the period from 1963 to 2002 highlighted substantial enhancements in survival, addressing both unilateral and bilateral cases [14]. Swift intervention prompted by timely detection plays a crucial role in achieving more favorable outcomes. While progress has been achieved, continuous research and initiatives to improve access to advanced care in developing nations are vital for ensuring every child has a meaningful opportunity for a healthy future. MacCarthy et al. conducted a comparative analysis spanning from 1963 to 1982 and from 1983 to 2002, revealing substantial enhancements in survival rates. Unilateral instances saw an increase from 85% to a remarkable 97%, while bilateral cases jumped from 88% to nearly perfect at 100% [15]. These data underscore the pivotal role of early identification in achieving positive results.

The management of retinoblastoma is complex, involving meticulously chosen strategies such as enucleation, radiotherapy, chemotherapy, laser photocoagulation, thermotherapy, and cryotherapy. Tailored care, accounting for variables such as the International Classification of Retinoblastoma (ICRB) staging, germline mutation status, psychosocial aspects, cultural perspectives, and institutional resources, remains crucial. The selection of treatment heavily relies on the ICRB classification, signifying the extent of tumor damage and influencing the choice of intervention. In recent times, chemoreduction, especially intravenous drug administration preceding other therapies, has emerged as a fundamental element in the management of retinoblastomas [16,17]. Ancona-Lezama et al. investigated modern methodologies, examining intravenous chemotherapy (IVC), intra-arterial chemotherapy (IAC), intravitreal chemotherapy (IVitC), and intracameral chemotherapy (IcamC). Cryotherapy, transpupillary thermotherapy (TTT), external beam radiotherapy (EBRT), and plaque radiotherapy are employed in consolidation treatments, often in conjunction with enucleation [18].

Chemotherapy, recognized as the primary eye-sparing approach for retinoblastoma, is administered through intravenous, intra-arterial, periocular, and intravitreal routes. Until 2010, IVC held precedence, utilizing vincristine, etoposide, and carboplatin to shrink tumors before focal therapies. IVC demonstrated remarkable efficacy, preserving nearly 100% of eyes in groups A, B, and C when combined with adjunctive therapies [19]. IAC competes with IVC in effectiveness for early retinoblastoma, achieving success rates approaching 100%. It serves as the primary treatment for specific unilateral cases and as secondary therapy for recurrent tumors, subretinal seeds, and vitreous seeds. IAC surpasses IVC in outcomes for advanced stages (groups D and E) [20-23]. Periocular chemotherapy strategically targets advanced bilateral cases, while IVitC offers a direct approach for persisting vitreous seeding. IVC remains indispensable for high-risk cases susceptible to metastatic dissemination [24]. The summary of treatment options for retinoblastoma is given in Table 1.

**Table 1:** Summary of treatment options for retinoblastoma

Treatment Approach	Description	Advantages	Disadvantages	References
Enucleation	Surgical removal of the eye	Offers definitive cure for advanced tumors	Loss of vision in affected eye	MacCarthy et al., 2009 [15]
Radiotherapy	Use of radiation to kill cancer cells	Can be effective for advanced tumors, preserves the eye	Risk of long-term side effects like vision loss and secondary tumors	MacCarthy et al., 2009 [15]
Laser photocoagulation	Use of focused laser light to destroy tumors	Minimally invasive, effective for small tumors	May not be effective for large tumors	MacCarthy et al., 2009 [15]
Transpupillary thermotherapy	Heating of tumor tissue	Minimally invasive, can be used for small tumors	May not be effective for large tumors	MacCarthy et al., 2009 [15]
Cryotherapy	Freezing of tumor tissue	Minimally invasive, effective for small tumors	May not be effective for large tumors	MacCarthy et al., 2009 [15]
Intravenous chemotherapy	Drugs delivered through a vein	Effective for early-stage tumors	Can have systemic side effects	Chung et al., 2015, Shields et al., 1997 [16, 17]
Intra-arterial chemotherapy	Drugs delivered directly to the tumor artery	Highly effective for early and later stage tumors, good for recurrent tumors and vitreous seeds, Less Systemic toxicity	May have side effects related to the blood vessels	Chung et al., 2015, Shields et al., 1997 [16, 17]
Periocular chemotherapy	Drugs delivered around the eye	Targets advanced bilateral tumors	Can have side effects related to the eye and surrounding tissues	Chung et al., 2015, Shields et al., 1997 [16, 17]
Intravitreal chemotherapy	Drugs delivered directly into the eye	Directly targets persistent vitreous seeding	May have side effects related to the eye	Chung et al., 2015, Shields et al., 1997 [16, 17]

## Review

Intravitreal therapy for retinoblastoma: a paradigm shift in treatment strategies

In recent years, IVitC has emerged as an innovative treatment, administering chemotherapy directly to the eye to address advanced retinoblastoma. During this period, the method has gained global acceptance, being adopted in multiple nations. Its effectiveness is evident, with certain centers viewing it as a groundbreaking strategy. Notably, there have been documented reductions in enucleation rates, indicating a departure from conventional treatments such as radiation and systemic chemotherapy. Despite these positive outcomes, skepticism persists in specific centers due to limited exposure, and ongoing discussions surround the treatment's long-term efficacy and potential drawbacks [25].

Intra-vitreal chemotherapy beyond vitreous seeds

The utility of intra-vitreal chemotherapy extends beyond eyes with vitreous seeds. Traditionally, IVitC has primarily targeted retinoblastoma tumors present in the vitreous (vitreous seeds). A study conducted by Abramson et al. delved into its efficacy beyond this scope, examining its potential to address the disease in other areas of the eye. Analyzing data from 56 eyes in 52 patients, they observed significant success. IVitC reduced tumors in an impressive 98% of cases, displaying minimal toxicity throughout the treatment course. While some recurrences occurred, particularly in retinal and subretinal locations, the overall outcomes are encouraging [26]. This indicates that IVitC could serve as a valuable additional therapy for preserving eyes in retinoblastoma patients, especially those with subretinal seeds or recurrent retinal tumors.

Targeted therapies paving the way: intravitreal melphalan

Advancements in retinoblastoma treatment are underway, especially in the development of targeted therapies, with a specific focus on intravitreal melphalan. This approach provides enhanced control over tumors and results in milder side effects compared to systemic alternatives. The study conducted by Shield et al. revealed promising outcomes, with cases treated using intravitreal melphalan demonstrating 100% eye preservation and no recurrence during a 15-month follow-up [27]. Vitreous seeds, recognized for their resistance to radiation and systemic chemotherapy, present a significant challenge; however, research by Munier et al. showcases successful results with intravitreal melphalan injections [28,29]. The potential to eradicate vitreous seeding, even in cases with prior treatments, positions intravitreal melphalan as an innovative treatment choice. Solana-Altabella et al. conducted a study aiming to establish and implement an inventive IVitC protocol for managing vitreous seeds in children with retinoblastoma. This involved the administration of melphalan injections and the evaluation of patient outcomes. Spanning from December 2014 to July 2018, the protocol averaged 3.3 cycles with standard 30 mcg doses of intravitreal melphalan. Encouragingly, the treatment yielded anticipated responses, effectively addressing vitreous seeding in three out of seven eyes, constituting 43% of total eyes with vitreous seeds [30]. Furthermore, in the challenging realm of advanced retinoblastoma with persistent vitreous seeds, a study involving 11 eyes demonstrated an exceptional 100% eye preservation with no recurrences even after a 15-month follow-up [31].

Research conducted by Shields et al. [32] and Kiratli et al. [33] highlights the significance of integrating both IVitC and IAC to advance retinoblastoma treatment, especially in advanced cases (group E). This combined approach shows reduced enucleation rates and improved eye preservation, emphasizing its effectiveness. Kiratli et al. further clarified the benefits of merging melphalan with topotecan, resulting in a significant decrease in enucleation rates and a slightly extended median survival [33].

In the initial stages, intraocular procedures were dismissed due to the potential spread of tumors beyond the eye. However, IVitC, particularly with melphalan, has emerged as a valuable option for addressing persistent vitreous seeds. An examination involving 27 eyes reveals encouraging results, with 78% achieving complete regression of seeds. Despite its relative safety, the study underscores the importance of careful consideration in treatment planning, highlighting side effects, primarily retinal toxicity. The findings suggest that intravitreal melphalan is a secure and effective treatment approach for retinoblastoma, enhancing the outcomes of procedures aimed at salvaging the eye [34].

The use of the intravitreal approach for chemotherapy in retinoblastoma has generated varied results. While some studies support its positive outcomes, others question its efficacy in tumor control [35]. Ongoing research is crucial to establishing the definitive role of IVitC in the comprehensive treatment strategy for retinoblastoma.

In conclusion, the evolving landscape of retinoblastoma treatment, marked by targeted therapies and innovative approaches, holds promise for improved outcomes and enhanced quality of life for affected children. Continued research and collaborative efforts globally are essential to refine and optimize these treatment modalities for the benefit of young patients facing this challenging diagnosis.

Adverse effects of local chemotherapeutic drugs: balancing safety and efficacy

In the treatment landscape of retinoblastoma, employing intra-vitreal chemotherapy raises various considerations about side effects. A notable consequence is the risk of tumor spread beyond the eye. A systematic analysis, amalgamating data from 14 studies on IVitC for retinoblastoma, reveals that, out of 1,304 injections administered to 315 eyes involving 304 patients, only one instance reported the extension of tumors beyond the eye. Another patient demonstrated a potential link between IVitC and metastatic disease. The combined findings suggest a rare incidence of tumor spread beyond the eye, amounting to a mere 0.007%. Crucially, within a subgroup of 61 patients undergoing IVitC with safety-enhancing injection techniques, no instances of tumor spread were observed. These findings underscore the viewpoint that the fear of tumor spreading should not discourage the use of IVitC for carefully selected retinoblastoma cases - those limited locally with no metastasis. This consideration becomes particularly pertinent when incorporated into a comprehensive approach focused on preserving the globe [31].

In comparison, intravitreal injections exhibit fewer local adverse effects and no identified systemic toxicity compared to IAC [36]. Shields et al.'s thorough examination, involving 192 eyes, sheds light on the local impacts of intravitreal melphalan, including pigment mottling (32%), focal cataracts (25%), transient vitreous hemorrhage (13%), hypotony (8%), optic disc edema (3%), and hemorrhagic retinal necrosis (3%). Multiple injections may lead to iris depigmentation and atrophy [37]. Rao et al.'s study suggests that topotecan could be a safer alternative to melphalan in pigmented retinoblastoma eyes with vitreous seeds, achieving 100% seed regression without reported ocular or systemic side effects [38].

In a review of 10 studies conducted by Smith et al., the ocular consequences of IVitC for retinoblastoma were meticulously investigated to provide valuable insights for enhanced patient care. This systemic evaluation revealed a total of 1,287 intravitreal injections administered to 306 eyes of 295 patients. The average follow-up period was 74.1 months, with 261 patients (88.5%) receiving standard melphalan IVitC doses ranging from 8 to 30 mcg. Ocular side effects were observed in 38 patients, with 17 categorized as significant and 21 as minor. The proportion of patients experiencing potentially significant ocular side effects after standard melphalan IVitC regimens was calculated at 0.031 (8/261). Among the significant cases, three patients experienced iris atrophy, while two each demonstrated chorioretinal atrophy and vitreous hemorrhage, and one had a retinal detachment. Importantly, five patients faced vision-threatening complications after substantial dose escalations (four with melphalan, one with thiotepa), three encountered issues linked to concurrent therapies, and one suffered a retinal detachment. For the 61 patients receiving IVitC through safety-enhancing injection techniques, all six significant side effects were either attributed to the therapeutic dose or complicated by concurrent treatments. The findings underscore that significant ocular complications following IVitC for retinoblastoma are infrequent [39]. Careful measures, such as a cautious injection technique with the injection site selected 2 O' clock hours away from the site of vitreous seeds, 3-3.5 mm away from the limbus at pars plana, and adherence to standard dosing regimens, can potentially reduce this risk [27,31].

In their assessment of ocular toxicity, Rao et al. observed an average decrease of 5.3 μV in electroretinography (ERG) response per melphalan injection. Factors influencing toxicity include injection proximity to IAC, eye pigmentation level, injection frequency, and the use of IAC. Despite these considerations, the study reports robust two-year survival rates - 94.2% ocular and 86.2% disease-free underscored the favorable risk-benefit profile [38].

Kiratli et al. proposed that administering intravitreal melphalan at 30-40 µg in one or two injections effectively addresses vitreous disease in 69.2% of eyes. Common side effects include vitreous hemorrhage (18%) and alterations in retinal pigment epithelium (8%) [40]. Suzuki et al.'s extensive retrospective analysis, involving 264 retinoblastoma eyes treated with intravitreal melphalan, reveals encouraging safety and efficacy outcomes. The risk of cancer spreading beyond the eye remains minimal, with fewer than 1.5% experiencing substantial side effects [41]. These findings highlight the optimistic trajectory of intravitreal melphalan as a secure and efficient therapeutic alternative, instilling hope for the preservation of vision in retinoblastoma cases with vitreous involvement. The summary of side effects is given below in Table 2.

**Table 2:** Summary of side effects of intravitreal chemotherapy

Side effects	Frequency	References
Pigment mottling	32%	Shields et al., 2016 [37]
Focal cataracts	25%	Shields et al., 2016 [37]
Transient vitreous hemorrhage	13-18%	Shields et al., 2016, Kiratli et al., 2017 [37, 40]
Hypotony	8%	Shields​​​​​​​ et al., 2016 ​​​​​​​[37]
Optic disc edema	3%	Shields​​​​​​​ et al., 2016 ​​​​​​​[37]
Hemorrhagic retinal necrosis	3%	Shields​​​​​​​ et al., 2016 ​​​​​​​[37]
Iris depigmentation and atrophy	After multiple injections, Extremely rare	Shields​​​​​​​ et al., 2016 ​​​​​​​[37]
Iris atrophy	0.01%	Smith​​​​​​​ et al., 2014 [39]
Chorioretinal atrophy	0.006%	Smith​​​​​​​ et al., 2014 ​​​​​​​[39]
Vitreous hemorrhage	0.006%	Smith​​​​​​​ et al., 2014 ​​​​​​​[39]
Retinal detachment	0.003%	Smith​​​​​​​ et al., 2014 ​​​​​​​[39]
Extraocular tumor spread	<1.5%	Suzuki at al., 2015 [41]
Retinal pigment epithelial alterations	8%	Suzuki at al., 2015 [41]

In summary, extraocular tumor spread with IVitC is a rare occurrence. Melphalan injection leads to a reduction in ERG response by approximately 5.3 μV per injection. While intravitreal melphalan is considered safe systemically, its impact on the retina is dose-dependent. Achieving the right equilibrium between efficacy and safety demands vigilant monitoring and thoughtful dose adjustments.

Balancing act: retinal impact and systemic safety

While holding promise for retinoblastoma treatment, the weekly administration of 30 µg melphalan via injections can have adverse effects on the retina. Clinical studies highlight a dose-dependent toxic impact, with each injection resulting in a decrease of ERG amplitudes by approximately 5.8 µV. This consistent finding, observed in both human and rabbit studies, underscores the necessity for meticulous monitoring, considering injection frequency and dosage [36]. Although intravitreal melphalan demonstrates systemic safety, the potential for retinal toxicity calls for a nuanced approach to strike a delicate balance between therapeutic efficacy and ocular safety.

In summary, IVitC has gained global acceptance, proving effective in reducing enucleation rates. Its efficacy in the whole eye has a success rate of 98%, suggesting its potential as an additional therapy for eye preservation. Intravitreal Melphalan, a targeted therapy, has shown promising outcomes with 100% eye preservation in certain cases. Combining IVitC and IAC in advanced cases proves effective, reducing enucleation rates. Concerns about IVitC include a rare risk of tumor spread and dose-dependent retinal toxicity, emphasizing the need for careful monitoring. Despite challenges, IVitC holds promise for improving outcomes in retinoblastoma, necessitating ongoing research and collaborative efforts for optimization and enhanced patient care.

## Conclusions

The landscape of retinoblastoma treatment is undergoing a transformative shift, with a dual focus on preserving vision and enhancing overall survival rates. While chemoreduction remains vital for high-risk cases, targeted therapies, notably intravitreal melphalan, are gaining prominence and exhibiting remarkable success in treating vitreous seeds and subretinal tumors. This progress raises the prospect of these targeted interventions potentially replacing conventional approaches such as radiation and systemic chemotherapy, particularly in advanced cases, as their combination proves to enhance efficacy.

The advent of minimally invasive techniques, exemplified by chemotherapy techniques and optimized dosing, is emerging as a crucial consideration for minimizing side effects associated with intravitreal therapies. However, the global perspective reveals stark disparities in survival rates between developed and developing nations. Bridging these gaps demands a multifaceted approach, including increased awareness, early diagnosis initiatives in resource-limited settings, improved access to advanced treatments, and collaborative efforts to address cultural and socioeconomic barriers to treatment adherence. In conclusion, the evolving narrative in retinoblastoma treatment offers promise for improved outcomes, and a brighter future for children diagnosed with this challenging disease hinges on sustained research, global collaboration, and ensuring equitable access to advanced therapies.

## References

[1] Stacey AW, Bowman R, Foster A, Kivelä TT, Munier FL, Cassoux N, Fabian ID (2021). Incidence of retinoblastoma has increased: results from 40 European countries. Ophthalmology.

[2] Shields JA, Shields CL (2008). Retinoblastoma: clinical and pathologic features. Intraocular Tumors: A Text and Atlas.

[3] Mehyar M, Mosallam M, Tbakhi A (2020). Impact of RB1 gene mutation type in retinoblastoma patients on clinical presentation and management outcome. Hematol Oncol Stem Cell Ther.

[4] Shields CL, Shields JA (2006). Basic understanding of current classification and management of retinoblastoma. Curr Opin Ophthalmol.

[5] Shields CL, Mashayekhi A, Au AK, Czyz C, Leahey A, Meadows AT, Shields JA (2006). The International Classification of Retinoblastoma predicts chemoreduction success. Ophthalmology.

[6] Broaddus E, Topham A, Singh AD (2009). Incidence of retinoblastoma in the USA: 1975-200. Br J Ophthalmol.

[7] Munier FL, Beck-Popovic M, Chantada GL (2019). Conservative management of retinoblastoma: challenging orthodoxy without compromising the state of metastatic grace. "Alive, with good vision and no comorbidity". Prog Retin Eye Res.

[8] Fabian ID, Abdallah E, Abdullahi SU (2020). Global retinoblastoma presentation and analysis by national income level. JAMA Oncol.

[9] Menon BS, Alagaratnam J, Juraida E, Mohamed M, Ibrahim H, Naing NN (2009). Late presentation of retinoblastoma in Malaysia. Pediatr Blood Cancer.

[10] Erwenne CM, Franco EL (1989). Age and lateness of referral as determinants of extra-ocular retinoblastoma. Ophthalmic Paediatr Genet.

[11] Naseripour M (2012). "Retinoblastoma survival disparity": the expanding horizon in developing countries. Saudi J Ophthalmol.

[12] Kivelä T (2009). The epidemiological challenge of the most frequent eye cancer: retinoblastoma, an issue of birth and death. Br J Ophthalmol.

[13] Meel R, Radhakrishnan V, Bakhshi S (2012). Current therapy and recent advances in the management of retinoblastoma. Indian J Med Paediatr Oncol.

[14] Jain M, Rojanaporn D, Chawla B, Sundar G, Gopal L, Khetan V (2019). Retinoblastoma in Asia. Eye (Lond).

[15] MacCarthy A, Birch JM, Draper GJ (2009). Retinoblastoma: treatment and survival in Great Britain 1963 to 2002. Br J Ophthalmol.

[16] Chung CY, Medina CA, Aziz HA, Singh AD (2015). Retinoblastoma: evidence for stage-based chemotherapy. Int Ophthalmol Clin.

[17] Shields CL, De Potter P, Himelstein BP, Shields JA, Meadows AT, Maris JM (1996). Chemoreduction in the initial management of intraocular retinoblastoma. Arch Ophthalmol.

[18] Ancona-Lezama D, Dalvin LA, Shields CL (2020). Modern treatment of retinoblastoma: a 2020 review. Indian J Ophthalmol.

[19] Murphree AL, Villablanca JG, Deegan WF 3rd (1996). Chemotherapy plus local treatment in the management of intraocular retinoblastoma. Arch Ophthalmol.

[20] Suzuki S, Yamane T, Mohri M, Kaneko A (2011). Selective ophthalmic arterial injection therapy for intraocular retinoblastoma: the long-term prognosis. Ophthalmology.

[21] Thampi S, Hetts SW, Cooke DL (2013). Superselective intra-arterial melphalan therapy for newly diagnosed and refractory retinoblastoma: results from a single institution. Clin Ophthalmol.

[22] Abramson DH, Fabius AW, Francis JH (2017). Ophthalmic artery chemosurgery for eyes with advanced retinoblastoma. Ophthalmic Genet.

[23] Shields CL, Manjandavida FP, Arepalli S, Kaliki S, Lally SE, Shields JA (2014). Intravitreal melphalan for persistent or recurrent retinoblastoma vitreous seeds: preliminary results. JAMA Ophthalmol.

[24] Shields CL, Lally SE, Leahey AM, Jabbour PM, Caywood EH, Schwendeman R, Shields JA (2014). Targeted retinoblastoma management: when to use intravenous, intra-arterial, periocular, and intravitreal chemotherapy. Curr Opin Ophthalmol.

[25] Abramson DH (2011). Chemosurgery for retinoblastoma: what we know after 5 years. Arch Ophthalmol.

[26] Abramson DH, Ji X, Francis JH, Catalanotti F, Brodie SE, Habib L (2019). Intravitreal chemotherapy in retinoblastoma: expanded use beyond intravitreal seeds. Br J Ophthalmol.

[27] Manjandavida FP, Shields CL (2015). The role of intravitreal chemotherapy for retinoblastoma. Indian J Ophthalmol.

[28] Munier FL, Gaillard MC, Balmer A, Soliman S, Podilsky G, Moulin AP, Beck-Popovic M (2012). Intravitreal chemotherapy for vitreous disease in retinoblastoma revisited: from prohibition to conditional indications. Br J Ophthalmol.

[29] Munier FL, Gaillard MC, Balmer A, Beck-Popovic M (2013). Intravitreal chemotherapy for vitreous seeding in retinoblastoma: recent advances and perspectives. Saudi J Ophthalmol.

[30] Solana-Altabella A, Valero S, Balaguer J (2020). Intravitreal melphalan therapy for vitreous seeds in retinoblastoma: Implementation and outcomes of a new chemotherapy protocol. J Oncol Pharm Pract.

[31] Smith SJ, Smith BD (2013). Evaluating the risk of extraocular tumour spread following intravitreal injection therapy for retinoblastoma: a systematic review. Br J Ophthalmol.

[32] Shields CL, Alset AE, Say EA, Caywood E, Jabbour P, Shields JA (2016). Retinoblastoma control with primary intra-arterial chemotherapy: outcomes before and during the intravitreal chemotherapy era. J Pediatr Ophthalmol Strabismus.

[33] Kiratli H, Koç I, Öztürk E, Varan A, Akyüz C (2020). Comparison of intravitreal melphalan with and without topotecan in the management of vitreous disease in retinoblastoma. Jpn J Ophthalmol.

[34] Yousef YA, Al Jboor M, Mohammad M (2021). Safety and efficacy of intravitreal chemotherapy (melphalan) to treat vitreous seeds in retinoblastoma. Front Pharmacol.

[35] Shields CL, Fulco EM, Arias JD (2013). Retinoblastoma frontiers with intravenous, intra-arterial, periocular, and intravitreal chemotherapy. Eye (Lond).

[36] Francis JH, Schaiquevich P, Buitrago E (2014). Local and systemic toxicity of intravitreal melphalan for vitreous seeding in retinoblastoma: a preclinical and clinical study. Ophthalmology.

[37] Shields CL, Douglass AM, Beggache M, Say EA, Shields JA (2016). Intravitreous chemotherapy for active vitreous seeding from retinoblastoma: Outcomes after 192 consecutive injections. The 2015 Howard Naquin Lecture. Retina.

[38] Rao R, Honavar SG, Sharma V, Reddy VA (2018). Intravitreal topotecan in the management of refractory and recurrent vitreous seeds in retinoblastoma. Br J Ophthalmol.

[39] Smith SJ, Smith BD, Mohney BG (2014). Ocular side effects following intravitreal injection therapy for retinoblastoma: a systematic review. Br J Ophthalmol.

[40] Kiratli H, Koç İ, Varan A, Akyüz C (2017). Intravitreal chemotherapy in the management of vitreous disease in retinoblastoma. Eur J Ophthalmol.

[41] Suzuki S, Aihara Y, Fujiwara M, Sano S, Kaneko A (2015). Intravitreal injection of melphalan for intraocular retinoblastoma. Jpn J Ophthalmol.

